# Evaluating dosimetric outcomes: a comparison of CyberKnife, VMAT FFF, and Helical Tomotherapy Radixact for treating localized prostate cancer

**DOI:** 10.1007/s11547-025-02046-3

**Published:** 2025-09-11

**Authors:** Gaetano Gagliardo, Marcello Serra, Gianluca Ametrano, Marco Galiero, Cecilia Arrichiello, Valentina Borzillo, Francesca Buonanno, Valentina d’Alesio, Rossella Di Franco, Paolo Muto

**Affiliations:** 1 Post Graduate School in Medical Physics, University of Naples Federico II, Naples, Italy; 2 Department of Radiation Oncology, Istituto Nazionale Tumori—IRCCS—Fondazione G. Pascale, Naples, Italy; 3 LB Servizi per le Aziende SrL, Rome, Italy

**Keywords:** Prostate cancer, VMAT, VMAT FFF, CyberKnife, Helical tomotherapy, Stereotactic body radiation therapy

## Abstract

Prostate cancer (PCa) remains one of the most frequently diagnosed malignancies in men globally, ranking second in prevalence, with increasing incidence anticipated due to aging populations. Radiotherapy has emerged as a cornerstone treatment for localized PCa with stereotactic body radiation therapy (SBRT), and it offers a promising approach by delivering highly conformal high-dose radiation in fewer fractions. This study compares the dosimetric outcomes of three SBRT techniques—CyberKnife (CK), Volumetric Modulated Arc Therapy with flattening filter-free mode (VMAT FFF), and Helical Tomotherapy Radixact (HT)—for localized PCa. The investigation evaluates dose distribution characteristics, sparing of organs at risk (OARs), and treatment delivery efficiency. Two patient groups with low-to-intermediate-risk localized PCa treated at the Istituto Nazionale Tumori—IRCCS Fondazione G. Pascale were included. Treatment plans for CK, VMAT FFF, and HT were retrospectively generated and analyzed using dosimetric metrics such as Conformity Index (CI), Homogeneity Index (HI), Gradient Index, and dose–volume constraints for OARs. Results revealed comparable target coverage across all techniques, with CK demonstrating superior dose conformity and VMAT FFF achieving shorter treatment times. HT and VMAT FFF are effective alternatives, particularly for centers without access to CK systems, though CK offered advantages in challenging anatomical scenarios. Findings emphasize the feasibility of VMAT FFF and HT as viable options for expanding SBRT accessibility, enabling high-quality treatment delivery in diverse clinical settings.

## Introduction

Prostate cancer (PCa) is the second most prevalent cancer among men, with over 1.4 million new cases and 397,430 deaths reported worldwide in 2022 [1]. In Europe, it is the most diagnosed cancer in men and stands as the third leading cause of cancer-related mortality in this population [2].

The risk of developing PCa is closely associated with age: a systematic review of autopsy studies indicated that the prevalence of PCa in individuals under 30 years old was 5% (95% confidence interval [CI]: 3–8%). This prevalence increased with an odds ratio (OR) of 1.7 (95% CI: 1.6–1.8) per decade, reaching 59% (95% CI: 48–71%) by the age of over 79 years [3]. Considering the high incidence of the disease and the increasing life expectancy, the number of individuals affected by this condition is projected to rise. Specifically, it is anticipated that approximately 2,300,000 new cases will be diagnosed by 2040 [4].

Currently, advancements in screening campaigns, early diagnosis, and technological progress enable the detection of malignancies at their early stages, facilitating prompt intervention and resulting in a low mortality rate [5, 6]. Patients with localized disease identified at an early stage and classified as having a low to intermediate risk of recurrence generally experience a favorable prognosis, with a 10-year overall survival rate of 99% [7].

For the management of PCa, various therapeutic approaches are available [8]. In addition to surgery, radiotherapy (RT) has become a well-established treatment modality for this condition. It provides outcomes in terms of overall survival and quality of life (QoL) that are comparable to those achieved with surgical intervention. The optimal treatment choice is determined by the tumor risk classification, the patient’s performance status, and, importantly, the patient’s preferences and evaluation of potential side effects [7, 8].

The primary modalities of RT for PCa include external beam radiation therapy (EBRT), this involves directing high-energy radiation from outside the body toward the tumor; brachytherapy, this technique entails the implantation of radioactive sources directly within or near the prostate gland; and radiopharmaceuticals, these are radioactive isotopes administered systemically through injections, which target and irradiate cancerous tissues [9].

In traditional EBRT, a typical dose per fraction is approximately 2 Gy. However, based on radiobiological principles and the linear-quadratic model, variations in the α/β ratios between the tumor and adjacent healthy tissues can significantly influence the fractionation schedule, tumor control probability (TCP), and Normal tissue complication probability (NTCP). In particular because of the notable disparity in α/β ratios between the tumor and the surrounding organs at risk (OARs), the use of hypofractionation is strongly encouraged [10–13]. Indeed, stereotactic body radiation therapy (SBRT)—which administers highly targeted, elevated doses of radiation to a tumor in fewer sessions than traditional radiotherapy—has emerged as the most prevalent method for the radiotherapeutic management of high risk localized PCa in low–intermediate risk [14–16].

This study continues the line of research conducted at the Istituto Nazionale Tumori-IRCCS Fondazione G. Pascale [17–20], and it aims to perform a dosimetric comparison among three different techniques for PCa radiotherapy. Specifically, the study will compare SBRT delivered using CyberKnife (CK) (CK is considered one of the main devices to perform SBRT), via Volumetric Modulated Arc Therapy with flattening filter-free (VMAT FFF) and Helical Tomotherapy (HT) Radixact. The comparison will evaluate dose distribution characteristics, OARs sparing, and treatment delivery time.

This analysis has two exploratory objectives: the first one is to evaluate the potential integration of HT into the clinical practice of the radiotherapy department at Istituto Nazionale Tumori-IRCCS Fondazione G. Pascale, as an alternative to the CK treatments localized PCa. The second objective is to demonstrate that VMAT FFF can effectively match the performance of the other two techniques, making it a viable option for healthcare facilities that lack CK or HT equipment, thereby in this case, reducing patient wait times.

## Materials and methods

### CyberKnife

The CyberKnife (CK, Accuray Inc., Sunnyvale, CA, USA) is a linear accelerator with an energy of 6 MeV, mounted on a robotic arm with six degrees of freedom, which results in a non-isocentric dose delivery approach. Additionally, it is equipped with static circular collimators ranging from 5 to 60 mm in diameter, as well as a dynamic IRIS collimator. Specifically, the accelerator is capable of being positioned at 100 distinct nodes, with each node accommodating up to twelve possible directions. This configuration allows for a total of 1200 unique beam entry angles. What distinguishes the CK in the field of RT is its ability to deliver up to 125% of the prescribed dose within the tumor volume while simultaneously achieving substantial sparing of the OARs [21].

The CK employs assistive and adaptive technology known as image-guided radiotherapy (IGRT). It features an image-guided system that aids in precise patient positioning and enables real-time monitoring of target movements during treatment [22].

The imaging system comprises two X-ray tubes mounted on the ceiling at 90 degrees to each other and angled 45 degrees relative to the patient’s axis. These X-ray tubes are paired with a set of silicon detectors (flat panels) positioned on the floor adjacent to the treatment bed. The nominal tube voltage ranges from 40 to 150 keV. The patient is imaged every 45 or 60 s, with the live images being digitized and compared to images synthesized from the patient’s CT data (digitally reconstructed radiographs, DRRs). This approach enables the detection of intra-fractional target shifts and facilitates automatic adjustment by the treatment manipulator during the delivery of treatment [21].

In the treatment of PCa, to accurately locate the tumor and monitor any positional changes during treatment, four gold fiducials are implanted into the prostate wall. The reconstruction of the prostate’s position using these fiducials enables real-time adjustments of the accelerator relative to the target [23–25].

The CK system is specifically designed for SBRT, and the results obtained in the treatment of PCa are currently favorable [26–30].

### VMAT FFF and clarity system

Volumetric Modulated Arc Therapy (VMAT) represents an advanced radiotherapy approach that enables the development of treatment plans with comparable or enhanced quality relative to fixed-field Intensity Modulated Radiation Therapy (IMRT), while simultaneously decreasing the duration of each treatment fraction [31, 32].

VMAT is a variant of IMRT where the gantry continuously rotates around the patient at varying speeds and dose rates. Unlike conventional IMRT, which uses fixed gantry angles, VMAT optimizes dose distributions through beam rotation and continuous adjustment of the radiation field shape by the multileaf collimator (MLC). Typically, for prostate treatments, one or two coplanar 360-degree arcs are sufficient to achieve an excellent treatment plan. [33–35].

The linear accelerator (LINAC) utilized in this study is the Elekta Versa HD, which is equipped with photon energies of 6, 10, and 15 MeV. However, in our Institute, for the purposes of VMAT dose delivery, only the 6 MeV photon energy setting is employed. The largest field size available for treatment is 40 × 40 cm^2^, delineated by a pair of stationary collimators which are positioned orthogonally to the MLC. The MLC consists of 80 pairs of tungsten leaves, each with a projected width of 5 mm at the isocenter.

Patients involved in this study were actually treated by VMAT, but the LINAC was equipped with the Clarity System (Elekta, Stockholm, Sweden), an ultrasound guidance system specifically approved for use with VMAT [36]. It integrates a transperineal ultrasound probe that enables real-time visualization of the prostate throughout treatment. This system can automatically pause treatment if the prostate shifts significantly from its planned position on the CT scan. It reconstructs three-dimensional images from two-dimensional ultrasound data. Due to the absence of the radiotherapist from the treatment room during procedures, the probe performs automatic scans using a motorized sweeping mechanism. This probe can complete a full 75° sweep in 0.5 s, causing minimal patient discomfort beyond a slight internal vibration. Consequently, the probe enhances the efficiency of verifying the positions of tumors and critical structures. The use of this technique has demonstrated a notable reduction in positional discrepancies compared to conventional treatment methods [36–38]. Furthermore, since this device increases the localization accuracy of the prostate gland, a smaller expansion from the clinical target volume (CTV) to the planning target volume (PTV) in contouring phase is allowed and therefore a lower dose to the OARs is expected.

To pursue the aim of this work, CK-like plans have to be computed, therefore, to achieve precise delivery of a high-dose therapeutic plan with a steep dose gradient within the tumor volume, the linear accelerator must operate in flattening filter-free (FFF) mode, meaning that the beam is delivered without the homogenizing filter. When a flattening filter is utilized, it modifies the beam profile by reducing central intensity and spreading the dose, leading to a nearly uniform distribution across the field. Additionally, the filter hardens the beam by attenuating lower-energy photons. In contrast, FFF mode produces a beam that is narrower and more intense centrally, which improves the ability to conform the dose to the target. This mode also increases the dose rate, allowing for shorter treatment times. Such characteristics are particularly advantageous in stereotactic therapies, where delivering precise and high doses is crucial for effective treatment and minimizing the impact of target movement [39].

**Fig. 1:**
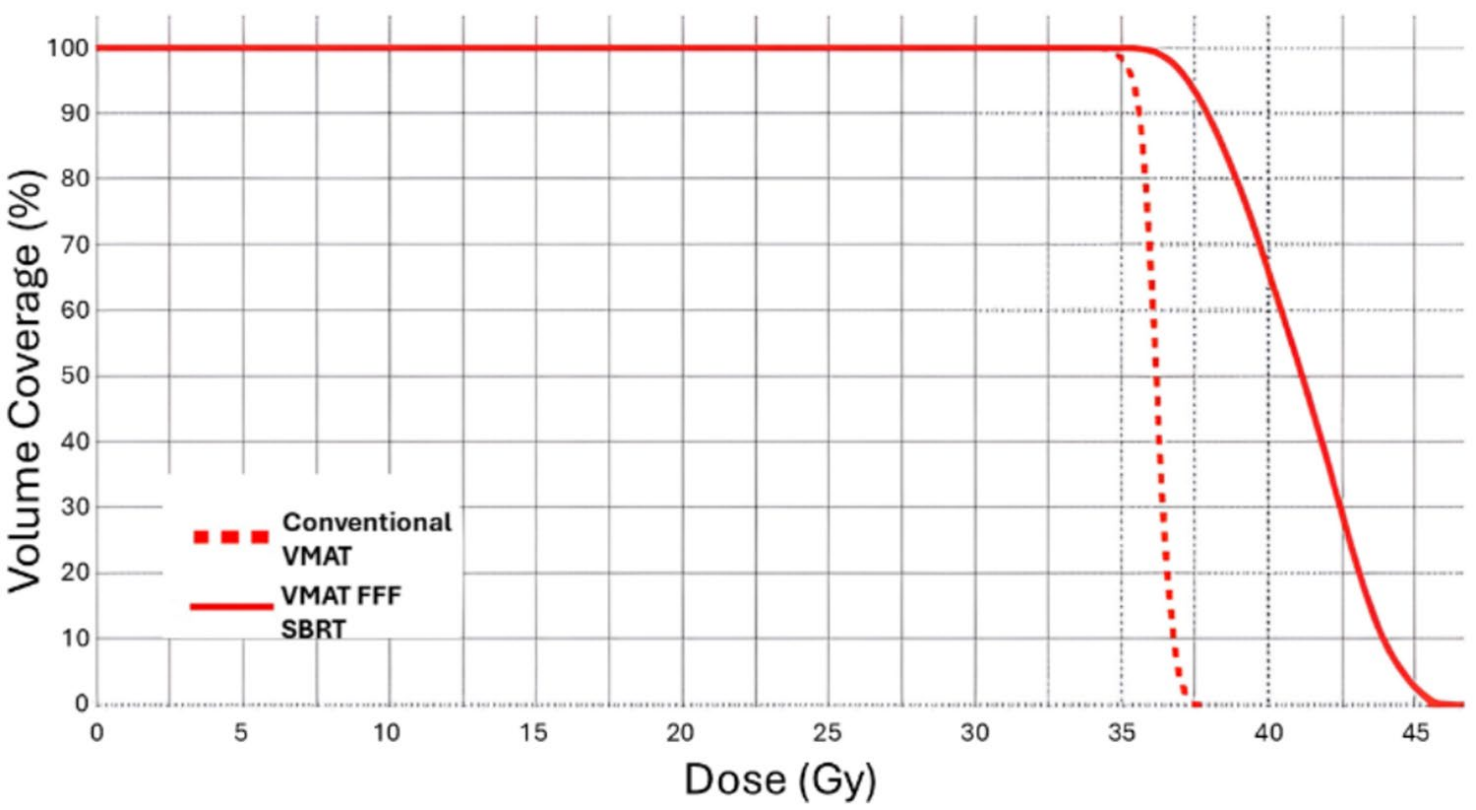
Comparison of DVHs for PTV between a conventional VMAT treatment and an SBRT treatment delivered using VMAT FFF. The difference between the two curves is clear

As previously outlined for the CK, SBRT is distinguished by two primary characteristics: the delivery of high doses to a relatively small and well-defined target volume (typically a few centimeters in diameter) across a minimal number of fractions (up to five); and a conical dose distribution profile, with the peak dose potentially reaching 125% of the prescribed dose at the geometric center of the target, and rapidly decreasing outside this region. In contrast, conventional radiotherapy treatments aim for a uniform dose across the target, with the dose not exceeding 110% of the prescribed value. Minor hotspots may be acceptable within the PTV. These conceptual differences lead to markedly different dose–volume histogram (DVH) profiles between stereotactic treatments and traditional VMAT. An optimal VMAT treatment plan is one where the DVH closely resembles a step function, maintaining the prescribed dose to the 100% of the PTV and dropping to 0% of the PTV volume beyond the prescription dose. Conversely, in a stereotactic plan, the descending portion of the DVH curve exhibits a more gradual slope, and the maximum dose can extend up to 125% of the prescribed dose (Fig. 1).

**Fig. 2:**
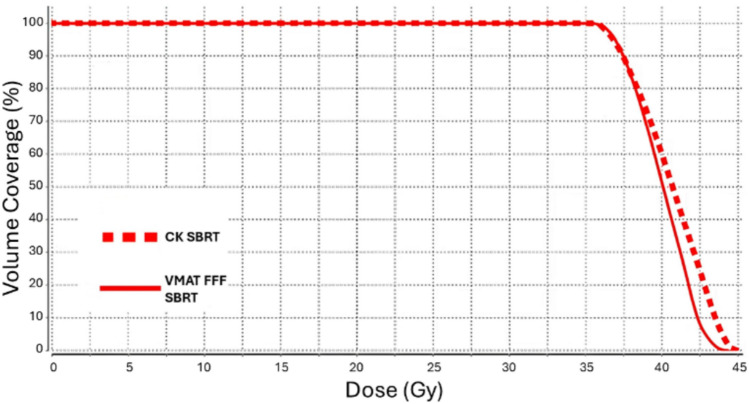
Comparison of DVHs for PTV between a CK SBRT plan and a VMAT FFF CK-like plan

To achieve the typical SBRT dose distribution using the VMAT, the FFF modality is not sufficient to guarantee the desired dose profile and OARs sparing, some adjustments are necessary in the planning phase: an auxiliary structure, equivalent to a few cubic centimeters within the central region of PTV volume was defined for each patient; the treatment-planning system (TPS) was then instructed to deliver a dose of 125% of the prescribed dose to 100% of this auxiliary structure’s volume. This approach compels the optimization algorithm to seek a dose distribution that peaks precisely at the desired location. In Fig. 2, to demonstrate that the goal of reproducing a CK-like plan was achieved, we present a comparison of the PTV DVHs for both the CK and VMAT FFF plans: they show a close overlap.

### Helical tomotherapy radixact

Helical tomotherapy (HT) is a form of IMRT that utilizes a coplanar arc configuration, incorporating a binary multileaf collimator (MLC) and a treatment field of 6 MeV fan beam shaped. This technique combines simultaneous table movement with a rotating radiation beam around the patient, thereby creating a helical treatment geometry. The HT system can image the patient by using either the treatment field or a lower energy X-ray tube, positioned orthogonally to the treatment filed. These component’s imaging capabilities enable the visualization of target areas before, immediately after the treatment and real time. This functionality replaces the traditional port films used in conventional radiotherapy, offering superior anatomical resolution [40].

Since its initial clinical deployment in 2002, a series of hardware and software enhancements have been integrated into HT. The first-generation Tomotherapy system, designated Hi-ART, was a specialized device engineered specifically for helical IMRT. The second-generation Tomotherapy system, known as Tomo-HD, introduced the capability for both fixed IMRT and helical IMRT, facilitated by the TomoDirect technology. Additionally, the TomoEDGE innovation enabled real-time adjustments to jaw width during dose delivery using a dynamic wedge, enhancing treatment precision. These advancements have been incorporated into the third-generation Tomo-HDA system, which integrates these technologies into a unified platform. The latest advancement in helical tomotherapy, developed by Accuray Inc., is Radixact, the fourth-generation system [41]. This system is currently in use at our Institute and is the focus of the dosimetric comparison presented in this article. Radixact features an upgraded gantry and a newly designed treatment-planning system. It has demonstrated that kilovolt-computed tomography (KV-CT) with the fast ClearRT technology significantly enhances both image quality and imaging speed compared to traditional megavoltage-computed tomography (MV-CT) [42]. Additionally, Radixact enables treatment with an elevated dose rate of 1000 cGy/min, surpassing the 850 cGy/min dose rate achievable with the Hi-ART, Tomo-HD, and Tomo-HDA systems [41]. Nowadays, Radixact is commercialized with the option to purchase a module (Synchrony) that allows for organ motion detection and correction during the treatment.

### Patients data and treatment planning

Two groups of patients with localized PCa, classified as low to intermediate risk and with a maximum prostate volume of 80 cc, were selected. All patients were treated at the Istituto Nazionale Tumori-IRCCS Fondazione G. Pascale from 2015 to 2019. Eligibility for enrolment was determined using the D’Amico risk classification system [43]. Low-risk patients included those with clinical stage T2a or lower, prostate-specific antigen (PSA) levels ≤ 10 ng/ml, and a Gleason score ≤ 6. Intermediate-risk patients were defined as having stage T2b-2c, PSA ≤ 20 ng/ml, and a Gleason score of 7 (3 + 4). Patients with a Gleason score of 7 (4 + 3) were excluded. The maximum allowable prostate volume for treatment was ≤ 90 cc. While androgen deprivation therapy (ADT) was not part of the standard protocol, a subset of patients received ADT for up to 3 months based on urological recommendations. No patients continued ADT during or after the radiotherapy course. Staging investigations included multiparametric magnetic resonance imaging (MRI) of the prostate; if contraindications to MRI were present, pelvic computed tomography (CT) was performed. Additionally, CT scans of the abdomen and chest, along with bone scintigraphy, were utilized to complete the TNM staging evaluation.

- *Group 1 (CK Group)*: Patients treated using the SBRT-CK technique (36.25 Gy in 5 fractions).
- *Group 2 (Clarity Group)*: Patients were imaged and treated with VMAT, but for sake of comparison with CK, a stereotactic treatment plan using VMAT FFF with the same prescription dose and fractionation as those used in SBRT-CK treatments was computed (36.25 Gy in 5 fractions).

Moreover, in order to have a broader comparison for each patient in both groups, a treatment plan was developed using the HT Radixact technique, with the same prescription dose and fractionation as those used for SBRT-CK treatments (36.25 Gy in 5 fractions).

The first group, referred to as the “CK Group,” consists of 10 patients (with target areas including the prostate or prostate and seminal vesicles) aged between 62 and 73 years, with an average prostate volume of 60 ± 20 cc. These patients were treated using SBRT-CK with an IGRT system that monitors the position of 4 internal gold radiopaque fiducials via X-ray motion tracking. The treatment was delivered using the IRIS collimator, with an average of 70 entry nodes, 270 total beams, and a mean dose rate of 800 MU/min. The CK treatment plans were developed using the dedicated “Precision” TPS from Accuray, employing the high-resolution “ray tracing” algorithm. Due to the delivery dose mode of the CK, within the inner parts of the target, a dose about the 125% of the prescription dose is achieved, therefore a dose of 36.25 Gy was prescribed as 80% of the maximum dose. The OARs considered in the planning were the bladder, rectum, and bowel. The most limiting constraints set for the inverse planning were V_37.5 Gy_ < 5 cc for the bladder and V_36.25 Gy_ < 5% for the rectum, utilizing two or three shells to achieve high-dose conformity to the PTV.

The second group, referred to as the “Clarity Group,” consists of 10 patients (with target areas including the prostate or prostate and seminal vesicles) aged between 70 and 81 years, with an average prostate volume of 50 ± 20 cc. These patients were treated starting in 2018 using traditional VMAT technique delivered by an Elekta Versa HD linear accelerator, with the Clarity ultrasound probe used for motion tracking. The treatment plans were developed using the Raystation TPS.

For the purpose of this work, for each patient in the Clarity Group, a treatment plan was computed using the TPS Monaco, creating an SBRT-VMAT FFF plan with the same prescription dose and fractionation as those used for the CK Group patients, i.e., 36.25 Gy in 5 fractions. The geometric configuration for all patients included two 360º arcs (clockwise (CW) and counterclockwise (CCW), with the collimator set at 10°, the patient table at 0º, and a segmentation into 120 parts per arc. The beam energy was 6 MV-FFF, and the dose calculation algorithm used was Montecarlo). The OARs considered in the planning were the bladder, rectum, and intestines.

The delineation of target volumes was conducted using a simulation CT scan integrated with prostate MRI fusion. For low-risk cases, the gross target volume (GTV) was defined as the prostate, while for intermediate-risk cases, it encompassed the prostate along with the proximal 2 cm of the seminal vesicles. The CTV was identical to the GTV. The PTV was created by expanding the CTV by 3 mm posteriorly and by 5 mm in all other directions. Organs at risk (OARs), including the rectum, bladder, penile bulb, femoral heads, bowel, testicles, and neurovascular bundle, were carefully contoured.

Table 1 presents the characteristics of the two patient groups in terms of target volume and OARs volumes.

**Table 1:** Target volume and OARs volumes for the two patient groups

Organs	Parameter	Group 1 (CK Group)	Group 2 (Clarity Group)
Prostate	Volume (cc)	60 ± 20	50 ± 20
	Range (cc)	36–86	24–80
Bladder	Volume (cc)	180 ± 170	210 ± 80
	Range (cc)	87–641	143–361
Bladder wall	Volume (cc)	60 ± 20	80 ± 20
	Range (cc)	48–91	61–123
Rectum	Volume (cc)	70 ± 20	70 ± 20
	Range (cc)	33–82	49–115
Rectal wall	Volume (cc)	30 ± 6	31 ± 7
	Range (cc)	15–37	23–48
Penis bulb	Volume (cc)	11 ± 4	6 ± 2
	Range (cc)	4–17	3–8

For both patient groups, treatment plans were re-planned using the HT Radixact technique, maintaining the same prescription dose and fractionation previously considered. The replanning was performed using the Accuray Precision TPS. The geometric configuration for all patients included a field width of 2.5 cm, a fixed pitch of 0.446, and a modulation factor of 2.000. The beam energy was set to 6 MV, and the dose calculation algorithm used was “VOLO.”

All plans, employing the three different techniques, were renormalized to ensure consistent target coverage (the minimum requirement was for the prescribed dose to encompass at least 95% of the PTV) and using the dose–volume constraints specified in Table 2 to assess their acceptability [44].

**Table 2:** Dose and volume constraints considered in the evaluation of treatment plans

Structure	Metric	Objective
Bladder	D_Max_ (Gy)	< 41.8 Gy
	D_1cc_ (Gy)	< 38 Gy
	V_37.5 Gy_ (cc)	< 5 cc
	V_37 Gy_ (cc)	< 10 cc
	V_100_ (%)	< 10%
	V_50_ (%)	< 40%
Bladder wall	D_Max_ (Gy)	< 105%
	D_10cc_ (Gy)	< 18.3 Gy
Rectum	D_Max_ (Gy)	< 38 Gy
	V_100_ (%)	< 5%
	V_36Gy_ (cc)	< 1 cc
	V_90_ (%)	< 10%
	V_80_ (%)	< 20%
	V_50_ (%)	< 50%
Rectal wall	D_Max_ (Gy)	< 105%
Bowel	D_Max_ (Gy)	< 28.5 Gy
Femoral head	D_Max_ (Gy)	< 24 Gy
	V_14.5 Gy_ (%)	< 5%
Penis bulb	V_29.5 Gy_ (%)	< 50%

All computed treatment plans were reviewed by experienced radiation oncologists to ensure they could be considered clinically acceptable.

### Comparative dosimetric study and statistical analysis

For each revised plan using the three different techniques, the DVH was extracted and analyzed. The mean values of dosimetric parameters, such as Conformity Index (CI), Homogeneity Index (HI), and Gradient Index (GI), dose to the target and OARs were calculated for each patient group, and the treatment duration as well. The calculated values were subsequently compared to perform a dosimetric comparison of the techniques, and the assessment of discrepancies was conducted using a Student’s t-test.

The Conformity Index (CI) measures the extent to which the dose distribution aligns with the target volume. In this study, we adopted the definition provided in [45], which is expressed as the ratio of the total volume of tissue receiving the prescribed dose to the volume of the target receiving the same dose, as outlined in (1):

1 $$CI=\frac{PIV}{TIV}$$

where $PIV$ is the prescription isodose volume, i.e., the total volume enclosed by the prescribed isodose, and $TIV$ is the target isodose volume, i.e., the volume of the target enclosed by the prescribed isodose.

The Homogeneity Index (HI) measures the uniformity of dose distribution within the target. However, stereotactic treatments inherently result in heterogeneous dose distributions within the target. Therefore, for statistical analysis, we utilized an adapted version [46, 47] of the HI that quantifies the peak dose within the PTV as described in (2):

2 $$HI=\frac{Dmax}{{RX}_{dose}}$$

where $Dmax$ and ${RX}_{dose}$ are the maximum dose and the prescribed dose to PTV, respectively.

The Gradient Index (GI) quantifies the rate of dose fall-off in a stereotactic treatment. It is defined [48, 49] as the ratio of the volume of a reference isodose,${PIV}_{\%}$, to the prescription isodose volume,$PIV$, as illustrated in ( 3):

3 $$GI\%=\frac{{PIV}_{\%}}{PIV}$$

Typically, ${PIV}_{50}$ is computed; it refers to the volume associated with an isodose value of 50% of the prescribed dose, and $GI50$=${PIV}_{50}/ PIV$.

## Results and discussion

In Tables 3 and 4 the mean values of CI, HI, GI, and treatment time (Δt) for the two different patient groups, across the various techniques, are reported. Statistical analyses were performed using a two-tailed Student’s t-test. The assumption of equal variances between groups was verified using Levene’s test prior to the t-test application. A *p*-value < 0.05 was considered statistically significant, and for each comparison we reported it in the corresponding tables.

**Table 3:** The mean values of CI, HI, GI25, GI50, GI75, and treatment time (Δt) compared via Student’s t-test for the two techniques HT Radixact and CyberKnife in Group 1 (CK Group)

Parameters	HT radixact	CyberKnife	ρ value
CI	1.1 ± 0.1	1.09 ± 0.04	< 0.001
HI	1.20 ± 0.01	1.24 ± 0.03	0.05
GI25	21 ± 3	24 ± 9	0.6
GI50	6 ± 1	5 ± 1	0.6
GI75	2.8 ± 0.4	2.7 ± 0.5	0.2
Δt (min)	8 ± 1	47 ± 9	0.001

**Table 4:** The mean values of CI, HI, GI25, GI50, GI75, and treatment time (Δt) compared via Student’s t-test for the two techniques HT Radixact and VMAT FFF in Group 2 (Clarity Group)

Parameters	HT Radixact	VMAT FFF	ρ value
CI	1.1 ± 0.1	1.0 ± 0.2	0.1
HI	1.14 ± 0.03	1.3 ± 0.1	0.02
GI25	21 ± 3	22 ± 4	0.05
GI50	4 ± 1	5 ± 1	0.05
GI75	2.5 ± 0.4	2.5 ± 0.3	0.07
Δt (min)	8 ± 1	3 ± 1	0.002

As observed, the HT Radixact technique and the VMAT FFF technique exhibit a higher average CI compared to the CK technique. This indicates that the CK technique provides superior dose conformity to the target. The average HI values indicate that the HT Radixact technique achieves a lower dose excursion, resulting in generally lower maximum dose values to the target for equivalent coverage. The HI is higher with the VMAT FFF technique (1.3 ± 0.1), which is significantly different from that obtained with the HT Radixact technique (1.14 ± 0.03) with a ρ value of 0.02. Regarding the average GI values for the three techniques, they are comparable, and the discrepancies among the parameters considered are not statistically significant, with significance defined as differences with a ρ value less than 0.05. This suggests that the dose gradient outside the PTV is very similar among the three techniques, making them practically equivalent in this regard as well. Finally, it is noteworthy that the average irradiation durations exhibit significant differences. For VMAT FFF, the duration is approximately fifteen times shorter compared to the CK technique. This is largely due to the use of the FFF mode, which increases the dose rate to 1300 MU/s, significantly reducing the delivery times.

In Tables 5 and 6, the mean values of the dose–volume constraints for OARs, aligned with those presented in Table 2, are provided for the two distinct patient groups across the different techniques.

**Table 5:** The mean values of the dose–volume constraints for OARs compared via Student’s t-test for the two techniques HT Radixact and CyberKnife in Group 1 (CK Group)

Structure	Objective	HT Radixact	CyberKnife	*p* value
Bladder	D_Max_ (Gy) < 41.8 Gy	(40 ± 2) Gy	(39 ± 2) Gy	0.6
	D_1cc_ (Gy) < 38 Gy	(38 ± 3) Gy	(37 ± 2) Gy	0.1
	V_37.5 Gy_ (cc) < 5 cc	(0.4 ± 0.5) cc	(1 ± 2) cc	0.162
	V_37 Gy_ (cc) < 10 cc	(0.7 ± 1.0) cc	(2 ± 2) cc	0.169
	V_100_ (%) < 10%	(1 ± 1) %	(2 ± 2) %	0.227
	V_50_ (%) < 40%	(35 ± 7) %	(37 ± 12) %	0.783
Bladder wall	D_Max_ (Gy) < 105%	(42.0 ± 0.1) Gy	(40 ± 2) Gy	0.08
	D_10cc_ (Gy) < 18.3 Gy	(37 ± 3) Gy	(32 ± 3) Gy	< 0.001
Rectum	D_Max_ (Gy) < 38 Gy	(40.0 ± 0.1) Gy	(37 ± 2) Gy	< 0.001
	V_100_ (%) < 5%	(0.01 ± 0.01) %	(0.2 ± 0.3) %	0.09
	V_36Gy_ (cc) < 1 cc	(0.1 ± 0.1) cc	(0.1 ± 0.2) cc	0.3
	V_90_ (%) < 10%	(5 ± 2) %	(3.5 ± 2.0) %	0.3
	V_80_ (%) < 20%	(11 ± 4) %	(10 ± 4) %	0.7
	V_50_ (%) < 50%	(41 ± 5) %	(35 ± 8) %	0.08
Rectal wall	D_Max_ (Gy) < 105%	(41.0 ± 0.1) Gy	(37.0 ± 0.5) Gy	< 0.001
Bowel	D_Max_ (Gy) < 28.5 Gy	(6 ± 1) Gy	(24 ± 10) Gy	0.4
Left Femoral head	D_Max_ (Gy) < 24 Gy	(16.0 ± 0.1) Gy	(15 ± 3) Gy	0.2
	V_14.5 Gy_ (%) < 5%	(0.7 ± 1.0) %	(1 ± 2) %	0.5
Right femoral head	D_Max_ (Gy) < 24 Gy	(16.0 ± 0.1) Gy	(15 ± 3) Gy	0.08
	V_14.5 Gy_ (%) < 5%	(0.9 ± 1.5) %	(1.5 ± 3.1) %	0.6
Penis bulb	V_29.5 Gy_ (%) < 50%	(3 ± 0) %	(1 ± 3) %	0.2

**Table 6:** The mean values of the dose–volume constraints for OARs compared via Student’s t-test for the two techniques HT Radixact and VMAT FFF in Group 2 (Clarity Group)

Structure	Objective	HT Radixact	VMAT FFF	*p* value
Bladder	D_Max_ (Gy) < 41.8 Gy	(41 ± 10) Gy	(41 ± 2) Gy	0.3
	D_1cc_ (Gy) < 38 Gy	(38.0 ± 0.1) Gy	(38 ± 1) Gy	0.1
	V_37.5 Gy_ (cc) < 5 cc	(1 ± 1) cc	(3 ± 2) cc	0.2
	V_37 Gy_ (cc) < 10 cc	(2 ± 2) cc	(4 ± 2) cc	0.2
	V_100_ (%) < 10%	(2 ± 1) %	(3 ± 1) %	0.1
	V_50_ (%) < 40%	(29 ± 3) %	(35 ± 10) %	0.2
Bladder wall	D_Max_ (Gy) < 105%	(42 ± 1) Gy	(41 ± 1) Gy	0.1
	D_10cc_ (Gy) < 18.3 Gy	(32 ± 4) Gy	(32 ± 4) Gy	0.1
Rectum	D_Max_ (Gy) < 38 Gy	(38 ± 1) Gy	(37 ± 1) Gy	0.2
	V_100_ (%) < 5%	(0.5 ± 0.7) %	(0.5 ± 0.7) %	0.5
	V_36Gy_ (cc) < 1 cc	(0.5 ± 0.6) cc	(0.5 ± 0.6) cc	0.7
	V_90_ (%) < 10%	(4 ± 3) %	(4 ± 3) %	0.8
	V_80_ (%) < 20%	(9 ± 5) %	(9 ± 4) %	0.3
	V_50_ (%) < 50%	(37 ± 9) %	(37 ± 9) %	0.6
Rectal wall	D_Max_ (Gy) < 105%	(40 ± 1) Gy	(37 ± 2) Gy	0.07
Bowel	D_Max_ (Gy) < 28.5 Gy	(0.02 ± 0.05) Gy	(0.02 ± 0.05) Gy	0.3
Left Femoral head	D_Max_ (Gy) < 24 Gy	(15.7 ± 1.0) Gy	(14 ± 2) Gy	0.05
	V_14.5 Gy_ (%) < 5%	(0.9 ± 1) %	(1 ± 2) %	0.1
Right femoral head	D_Max_ (Gy) < 24 Gy	(15.6 ± 1) Gy	(13 ± 2) Gy	0.02
	V_14.5 Gy_ (%) < 5%	(0.8 ± 2) %	(0.2 ± 0.5) %	0.4
Penis bulb	V_29.5 Gy_ (%) < 50%	(23 ± 26) %	(13 ± 18) %	0.003

For certain OARs, in some patients, neither CK, VMAT FFF, nor HT Radixact plans meet the prescribed dose constraints. This is attributed to the anatomical location of the prostate, whose walls, in some patients, are almost in direct contact with those of the bladder. Consequently, the PTV, being an expansion of the target (tumor volume), presents regions of overlap with adjacent organs. This configuration, combined with the high dose values prescribed for the target in stereotactic treatment, makes it extremely challenging to stay within the maximum dose limits. However, when considering the mean values of the dosimetric parameters, all techniques can be said to meet most of the prescribed dose constraints.

In the comparison between HT Radixact and CK, only the constraints D_10cc_ (Gy) on the bladder wall, D_Max_ (Gy) on the rectum, and D_Max_ (Gy) on the rectal wall show significant differences, with the CK technique being more conservative in all three cases, ensuring a lower dose. In fact, as shown by the average values in Table 5, the constraints D_Max_ (Gy) < 38 Gy for the rectum and D_Max_ (Gy) < 105% for the rectal wall are not satisfied with the HT Radixact technique. Additionally, the constraint D_1cc_ (Gy) < 38 Gy for the Bladder is at the threshold, although no significant differences are observed when compared to the CK technique. The constraints on the bladder wall, however, are not met by either technique.

In the comparison between HT Radixact and VMAT FFF, only the D_Max_ (Gy) constraint on the right femoral head and V_29.5 Gy_ (%) on the penis bulb show significant differences between the two techniques, but with both they are below the threshold value. In this case, the VMAT FFF technique is also more conservative than HT Radixact, resulting in lower dose values. As with previous comparisons, the constraints on the bladder wall are not met by either technique. Other constraints worth noting include D_1cc_ (Gy) < 38 Gy for the Bladder, which is at the threshold for both techniques, and D_Max_ (Gy) < 38 Gy on the rectum and D_Max_ (Gy) < 105% on the rectal wall, which are violated by the HT Radixact technique, though without significant differences compared to VMAT FFF.

The authors acknowledge that all the results presented in this study may be influenced by the relatively small patient cohort, which is primarily due to the strict inclusion criteria required for patients to undergo this type of treatment. However, we would like to point out that, given the statistical significance observed in the various tests, we believe the considerable difference in treatment duration between CyberKnife and the other two techniques cannot simply be attributed to more favorable anatomical conditions for the latter ones or to mere statistical fluctuations. Furthermore, our findings are consistent with those reported in other studies, thus providing confirmation of our results through independent patient cohorts. Nonetheless, further analyses with larger datasets are planned, in order to strengthen the robustness of the results or to highlight specific patient anatomical conditions in which one technique may demonstrate superior performance in terms of the dosimetric indices presented in this work.

## Conclusions

SBRT is considered one of the most effective options for localized prostate cancer. Presently, at the Radiotherapy Department of the Istituto Nazionale Tumori-IRCCS Fondazione G. Pascale, this goal is achieved delivering 36.25 Gy in 5 fractions by means of the CK.

The literature provides strong support for the use of SBRT in the treatment of prostate cancer. Specifically, a multicenter, international phase 3 randomized controlled trial is currently underway to evaluate whether hypofractionated SBRT offers a therapeutic advantage over prostatectomy or conventional radiotherapy in men with organ-confined prostate cancer. The preliminary results are promising, and we recommend that SBRT treatment will be made accessible to all patients who need it and it will be incorporated as a standard of care [15].

In this context, to manage patient flow more efficiently and flexibly, it is of clinical interest to explore the possibility of delivering stereotactic prostate treatments using the HT Radixact and the VMAT FFF, therefore in the latter case employing a “standard” linear accelerator for treatment delivery. Such a result would be highly significant and beneficial, as it would enable the extension of SBRT treatment to a larger number of patients nationwide, particularly those treated at radiotherapy centers not equipped with CK or HT systems which currently represent the vast majority. Another important aspect is the non-invasive nature of VMAT FFF compared to the internal fiducial marker implantation required for CK imaging, which imposes additional patient stress and radiation exposure beyond the treatment dose itself.

In this regard, the dosimetric differences between the techniques highlighted in this study were found to be small and clinically insignificant, primarily related to the different dose delivery methods of the CK system compared to VMAT FFF and HT Radixact. Specifically, the CK and VMAT FFF techniques tend to be more conservative compared to HT Radixact, delivering lower doses to the OARs and more consistently meeting dose constraints. Regarding dose distribution, while the CK technique generally conforms better to the target, the VMAT FFF technique achieves greater dose variation within the target. However, the discrepancies between the techniques are not significant enough to clearly favor one over the others. The dose fall-off outside the PTV is equivalent for the techniques, as indicated by the mean values of the GI, which show no significant differences. However, we want also notice that the HT Radixact plans are dosimetrically consistent between the two sets of patients, except for the penis bulb. In fact, the presence of the perineal probe, in the clarity group set, reduces the distance between the prostate and the penis bulb, and as result, the latter receives a higher dose. Another interesting point that emerges from the data is the maximum dose received by the bowel when CK is adopted. CK is a non-isocentric technique, and one of its strengths is the ability to target the tumor from angles that are not achievable for other devices. This flexibility results in significant variability among treatments plans, adapting more to the patients’ anatomy. Therefore, in some cases, clinically acceptable dose spikes may have occurred in the bowel, leading to a higher average dose value but also a larger standard deviation.

This study reinforces the findings reported in extensive literature comparing rotational techniques and CK, which consistently yield highly promising outcomes and show general agreement across different datasets [17, 18, 50, 51]. In the articles previously cited, the CI values obtained for the described techniques are consistent, within the limits of error, with those achieved in the present study. Regarding HI values, not all articles in literature calculate this parameter using the definition proposed in this work. An interesting observation is that, in none of the four studies, VMAT is the technique with the largest dose excursion. This observation can be attributed to the use of the VMAT FFF technique in this study, which is particularly advantageous for small targets like prostate. The VMAT FFF approach excels in dose modulation for such targets and allows for a wider dose range, similar to what is achieved in CK stereotactic treatments. This characteristic contributes to a higher HI. The absence of beam filtering allows for a significant dose escalation relative to the prescription dose and beam on time reduction.

Regarding the dose values for the OARs, there are differences compared to the cited studies, primarily due to the use of different target coverage volumes. However, the trend observed in our work aligns with the existing literature, confirming that the most challenging constraints to meet are those for the bladder and bladder wall, as these structures often overlap with the target. Similarly, the maximum dose constraints for the rectum also pose significant challenges.

Another aspect worth focusing is the beam on time for the different technologies: CK has significantly and undoubtedly longer treatment times. Plans computed in this study made use of IRIS collimator; it is composed of two hexagonal diaphragms designed to produce nearly circular beams, corresponding to the nominal field sizes provided by fixed collimators. In our Institute has been commissioned a new CK equipped with an MLC collimator. It is expected that the advantages of CK treatment can be combined with those of VMAT FFF ones; therefore, next studies will be aimed to the comparison between prostate treatment with CK IRIS and MLC collimator in order to evaluate the quality of the plans and required time for the dose delivery.

In this context, an emerging technology that warrants attention is the MR-Linac, which integrates real-time magnetic resonance imaging during treatment delivery [52, 53]. This unique capability provides advantages in dose optimization, reduction of side effects, and immediate adaptation to anatomical variations occurring during treatment. Studies in the literature indicate that SBRT treatments guided by MRI imaging can lead to substantial sparing of OARs [54, 55]. Due to differences in treatment protocols, a direct comparison between our results and those from MR-Linac-based studies is not feasible. In studies involving MR-Linac, it is generally accepted that the maximum dose delivered to the PTV does not exceed 107% of the prescribed dose. In contrast, our approach involves administering doses above 120% to replicate a CyberKnife-like treatment. Consequently, further research is required to assess the comparative effectiveness of MR-Linac in relation to existing techniques such as CyberKnife, VMAT FFF, and HT Radixact for SBRT.

In conclusion, HT Radixact and VMAT FFF are viable alternative for the SBRT of localized prostate cancer to the CK, provided there is a lesion monitoring, since it allows for tight margins in prostate expansion during treatment planning and double check when the dose is delivered, consequentially also wider OARs sparing is achieved. That being said, CK remains the primary tool for SBRT, especially in more challenging cases, allowing for extremely complex beam paths.

## Data Availability

The raw data are available at https://zenodo.org/records/15395232.
